# Case Report: Bilateral coronary ostial spasm triggered by iatrogenic thyrotoxicosis mimicking left main coronary artery disease

**DOI:** 10.3389/fcvm.2026.1814778

**Published:** 2026-04-10

**Authors:** Han Ra Choi, Jae Seok Bae, Yun-Ho Cho, Jeong Yoon Jang, Jeong Rang Park, Min Gyu Kang, Kye-Hwan Kim, Jin-Yong Hwang, Jong-Hwa Ahn

**Affiliations:** 1 Division of Cardiology, Department of Internal Medicine, Gyeongsang National University School of Medicine and Gyeongsang National University Changwon Hospital, Changwon, Republic of Korea; 2 Department of Internal Medicine, Gyeongsang National University School of Medicine and Gyeongsang National University Hospital, Jinju, Republic of Korea

**Keywords:** beta-blocker, bilateral coronary ostial stenosis, coronary vasospasm, left main coronary artery, ST-segment elevation, thyrotoxicosis

## Abstract

**Background:**

Thyrotoxicosis is associated with enhanced sympathetic activity and increased coronary vasomotor reactivity. However, bilateral coronary ostial spasm mimicking critical left main coronary artery disease is rare.

**Case summary:**

A 61-year-old woman presented with a 3-day history of exertional chest pain. She had undergone total thyroidectomy 18 years earlier and was receiving levothyroxine 75 μg daily. One month prior, carvedilol 6.25 mg twice daily had been initiated for palpitations. Treadmill testing demonstrated ST-segment elevation in aVR and anterior leads with reciprocal ST depression in inferior leads. Emergent coronary angiography revealed severe ostial narrowing of both the left main (LM) and right coronary artery (RCA), raising suspicion of critical multivessel disease. However, complete resolution of both lesions following intracoronary nitrate administration confirmed bilateral coronary vasospasm. Laboratory evaluation showed suppressed TSH (0.07 mIU/L) with markedly elevated free T4 and free T3 levels, consistent with iatrogenic thyrotoxicosis. Levothyroxine dose reduction, discontinuation of carvedilol, and initiation of calcium channel blocker therapy resulted in complete symptom resolution.

**Conclusion:**

Iatrogenic thyrotoxicosis may precipitate severe bilateral coronary ostial spasm that mimics obstructive coronary artery disease. Recognition of nitrate responsiveness and careful *β*-blocker selection are critical to avoid unnecessary revascularization.

## Introduction

Thyrotoxicosis exerts profound cardiovascular effects, including increased heart rate, enhanced myocardial contractility, reduced systemic vascular resistance, and heightened sympathetic activity (1). Excess thyroid hormone upregulates *β*-adrenergic receptor expression and amplifies catecholamine sensitivity, leading to significant alterations in coronary vasomotor tone (1, 2). While tachyarrhythmias and high-output heart failure are well-recognized manifestations of hyperthyroidism, coronary vasospasm is less commonly appreciated. Although the exact prevalence of coronary vasospasm in hyperthyroid patients is not well established, prior case series have suggested that thyroid hormone excess may increase susceptibility to vasospastic angina, with symptomatic improvement observed after restoration of euthyroidism (3, 4). Bilateral coronary ostial involvement is particularly uncommon, with only limited case reports described in the literature.

Coronary vasospastic angina, also known as variant angina, is characterized by transient coronary artery constriction resulting in myocardial ischemia and reversible ST-segment elevation (5). Although vasospasm typically affects a single epicardial vessel, multivessel or bilateral ostial involvement is rare and may mimic severe left main or multivessel obstructive coronary artery disease (6).

Electrocardiographically, ST-segment elevation in lead aVR accompanied by diffuse ST depression is classically associated with left main coronary artery disease or severe multivessel ischemia (7). This pattern often prompts urgent invasive evaluation due to concern for catastrophic coronary obstruction.

*β*-blockers are widely used in hyperthyroid patients to control adrenergic symptoms (8). However, non-selective *β*-blockade may theoretically exacerbate coronary vasospasm by attenuating *β*₂-mediated vasodilation and permitting unopposed *α*-adrenergic vasoconstriction (9). We report a case of iatrogenic thyrotoxicosis presenting as exercise-induced ST-segment elevation and angiographically apparent bilateral coronary ostial stenosis, which was completely reversible with nitrate administration.

## Case description

A 61-year-old woman presented with substernal squeezing chest pain aggravated by exertion for three days. She had a history of hypertension and dyslipidemia, treated with candesartan 16 mg daily and rosuvastatin 20 mg daily.

She had undergone total thyroidectomy for thyroid cancer 18 years earlier and had been maintained on levothyroxine 75 μg daily. One month before presentation, carvedilol 6.25 mg twice daily had been initiated for palpitations. She denied any prior history of exertional angina, syncope, or documented ischemic heart disease. She had not undergone prior coronary imaging or invasive cardiac evaluation, and her functional status before the recent episode had been stable without exercise limitation.

**Figure 1:**
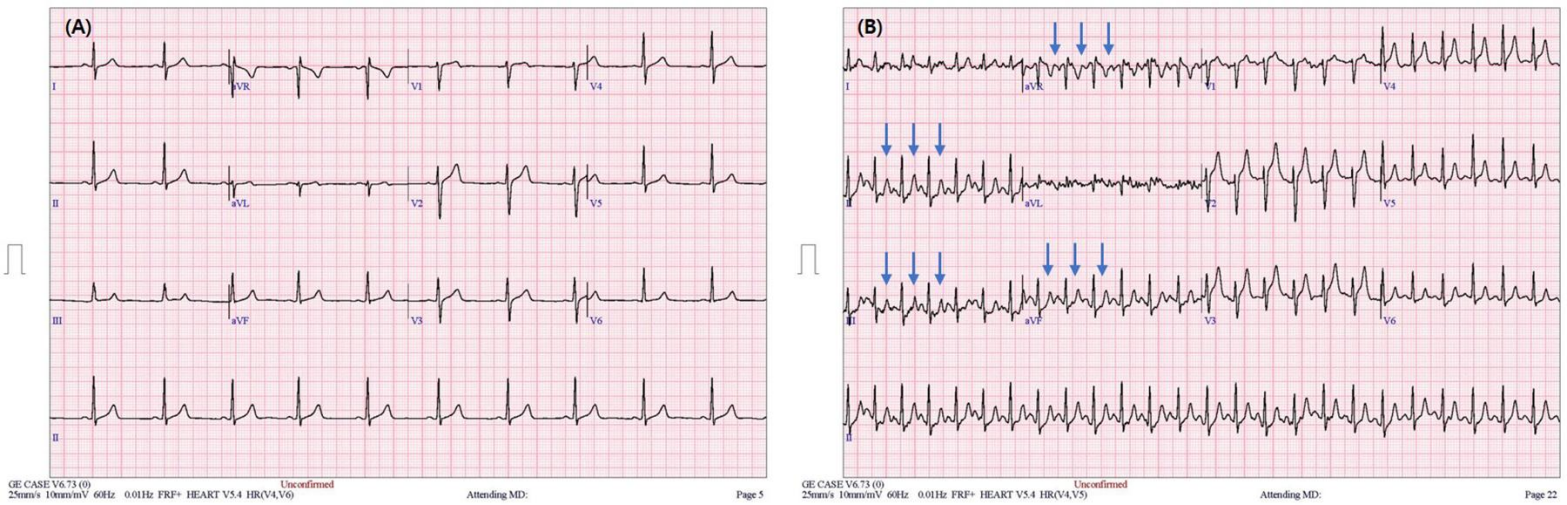
Treadmill electrocardiogram showing ST-segment elevation in aVR and anterior leads with reciprocal ST depression in inferior leads. **(A)** Baseline electrocardiogram prior to exercise testing showing normal sinus rhythm without significant ST-segment or T-wave abnormalities. **(B)** ST-segment elevation in aVR with widespread ST depression during exertion, a pattern classically associated with left main coronary artery obstruction but in this case attributable to dynamic bilateral coronary ostial spasm. Arrows indicate ST-segment elevation in lead aVR and reciprocal ST-segment depression in the inferior leads.

Baseline electrocardiography was normal. During stage 2 of treadmill testing, she developed chest discomfort accompanied by ST-segment elevation in aVR and anterior leads, with reciprocal ST depression in inferior leads (Figure 1). The test was terminated immediately.

## Diagnostic assessment

**Figure 2:**
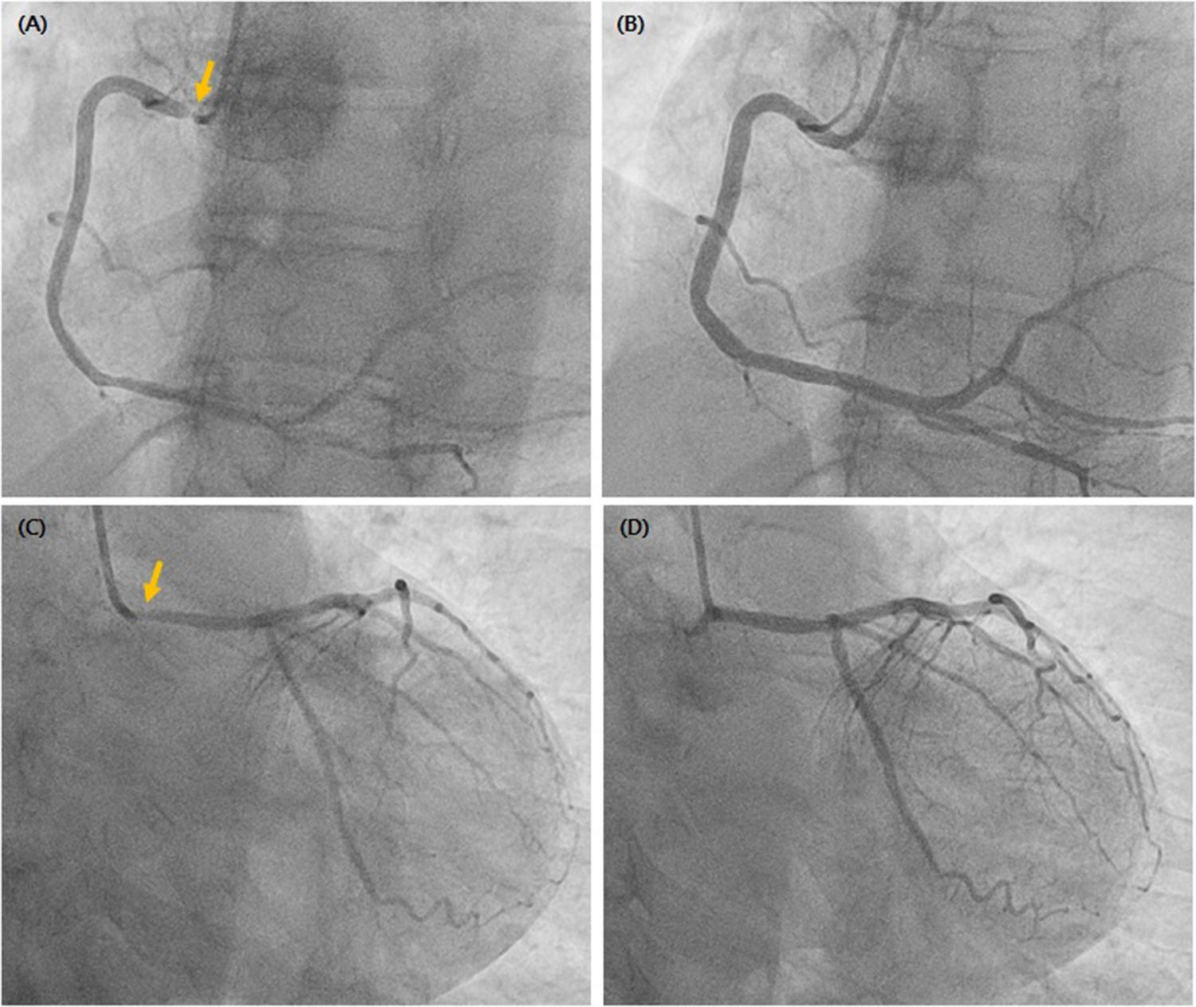
Coronary angiography. **(A)** Severe ostial stenosis of the right coronary artery (arrow). **(B)** Resolution after intracoronary nitrate. **(C)** Severe ostial stenosis of the left main coronary artery (arrow). **(D)** Resolution after intracoronary nitrate. Images were obtained in standard angiographic projections (right anterior oblique for RCA and left anterior oblique caudal view for LM). The ostial segments are explicitly indicated by arrows.

Given concern for left main coronary artery disease, emergent coronary angiography was performed. Severe ostial narrowing of the right coronary artery (RCA) and the left main (LM) coronary artery was observed (Figures 2A, C). Intracoronary nitrate (200 μg) was administered sequentially into the RCA and LM coronary artery. Within approximately 30–60 s, complete resolution of the ostial narrowing was observed, with restoration of normal luminal caliber and TIMI 3 flow (Figures 2B, D). This finding was consistent with bilateral coronary ostial spasm.

Laboratory testing revealed the following:

- TSH: 0.07 mIU/L (reference 0.55–4.78)
- Free T4: 3.34 ng/dL (reference 0.89–1.76)
- Free T3: 11.78 pg/mL (reference 2.3–4.20)
- Thyroglobulin antibody: 31.2 IU/mL (reference 0.0–4.50)
- Anti–thyroid peroxidase antibody: < 0.1 IU/mL (reference 0.0–13.80)
- TSH receptor antibody: < 0.80 IU/L (reference 0.0–1.75)
- High-sensitivity troponin I: 159.66 pg/mL (reference 2.5–38.64)

The suppressed TSH with markedly elevated free T4 and T3 levels in the absence of TSH receptor antibodies suggested iatrogenic thyrotoxicosis rather than autoimmune hyperthyroidism.

## Therapeutic intervention and follow-Up

Levothyroxine dose reduction and calcium channel blocker therapy were initiated, and carvedilol was discontinued. At 3-month follow-up, thyroid function tests demonstrated normalization of TSH levels. The patient remained asymptomatic without recurrence of anginal symptoms or ischemic electrocardiographic changes. Follow-up electrocardiography showed no residual ST-segment abnormalities, and no additional hospitalizations or emergency visits for cardiac symptoms occurred during the follow-up period.

## Discussion

This case illustrates several clinically important considerations regarding the interaction between thyroid hormone excess and coronary vasomotor function.

## Thyrotoxicosis and coronary vasomotor reactivity

Thyroid hormones increase myocardial oxygen demand and potentiate sympathetic signaling through genomic and non-genomic mechanisms (1, 2). Experimental and clinical studies have demonstrated that hyperthyroidism enhances vascular smooth muscle responsiveness to catecholamines and may impair endothelial-dependent vasodilation (2, 7). In addition to augmented sympathetic activity, endothelial dysfunction and increased vascular smooth muscle reactivity may contribute to coronary vasospasm in hyperthyroid states. Heightened catecholamine sensitivity further amplifies coronary vasomotor instability, underscoring the multifactorial nature of this condition.

Coronary vasospasm associated with hyperthyroidism has been reported, though it remains an uncommon presentation (3). In many cases, ischemic symptoms resolve following correction of thyroid hormone excess, underscoring a causal relationship (3, 4). In the present patient, markedly suppressed TSH with elevated free T4 and free T3 levels, in the absence of TSH receptor antibodies, supported the diagnosis of iatrogenic thyrotoxicosis due to levothyroxine replacement. The modest elevation in high-sensitivity troponin was interpreted as transient myocardial injury secondary to dynamic vasospasm, consistent with demand ischemia, rather than myocardial infarction due to fixed coronary obstruction.

## Bilateral coronary ostial spasm mimicking left main disease

The angiographic finding of severe ostial narrowing in both the left main and right coronary arteries raised immediate concern for critical obstructive disease. However, complete resolution following intracoronary nitrate administration confirmed a dynamic vasospastic process.

Bilateral ostial spasm is particularly dangerous because it can produce global subendocardial ischemia and electrocardiographic patterns typically associated with left main disease (7, 10). ST elevation in aVR with diffuse ST depression reflects widespread ischemia and is commonly linked to severe multivessel coronary obstruction (7). Recognition of nitrate reversibility is therefore essential to avoid unnecessary percutaneous or surgical revascularization.

## *β*-Blocker selection in hyperthyroid patients

*β*-blockers are first-line therapy for symptomatic hyperthyroidism (8). However, their effect on coronary vasomotion is complex. *β*₂-adrenergic receptors mediate coronary vasodilation, whereas *α*-adrenergic stimulation promotes vasoconstriction (9). Non-selective *β*-blockade may reduce *β*₂-mediated vasodilatory tone and allow relative *α*-adrenergic dominance, potentially exacerbating coronary spasm (6, 9). In contrast, *β*1-selective agents may theoretically exert less influence on coronary vasomotor tone in this setting, although direct comparative evidence remains limited. Therefore, careful *β*-blocker selection should be considered in hyperthyroid patients when vasospastic angina is suspected.

Although carvedilol possesses additional *α*₁-blocking properties, it remains a non-selective *β*-blocker and may not fully prevent dynamic vasoconstriction in susceptible individuals. In this patient, the recent initiation of carvedilol in the setting of untreated thyrotoxicosis may have contributed to the severity of bilateral coronary vasospasm.

After levothyroxine dose reduction, discontinuation of carvedilol, and initiation of calcium channel blocker therapy, the patient experienced complete resolution of symptoms without recurrence. Calcium channel blockers remain the cornerstone of vasospastic angina management (5).

## Conclusion

Iatrogenic thyrotoxicosis can precipitate severe bilateral coronary ostial spasm mimicking left main coronary artery disease. Exercise-induced ST-segment elevation with apparent critical ostial stenosis should prompt consideration of dynamic vasospasm, particularly in patients with biochemical hyperthyroidism. Intracoronary nitrate testing is essential to distinguish spasm from fixed obstruction. Careful *β*-blocker selection and prompt correction of thyroid hormone excess are critical for optimal management.

## Patient perspective

The patient reported significant anxiety when informed of the possibility of severe coronary artery disease. She expressed relief upon learning that the condition was reversible and related to thyroid hormone imbalance. She reported improved quality of life after medication adjustment.

## Data Availability

The original contributions presented in the study are included in the article/supplementary material, further inquiries can be directed to the corresponding author.
